# Predictors of long-term survival prior to permanent pacemaker implantation in octogenarians or older

**DOI:** 10.1007/s40520-018-1044-4

**Published:** 2018-09-27

**Authors:** Chi-Wen Cheng, Chao-Hung Wang, Wei-Siang Chen, Chun-Chieh Wang, Wen-Jin Cherng

**Affiliations:** 1 Heart Failure Research Center, Division of Cardiology, Department of Internal Medicine, Chang Gung Memorial Hospital, 222 Mai Chin Road, Keelung, Taiwan, ROC; 2 Chang Gung University College of Medicine, Taoyuan, Taiwan, ROC; 3 Division of Cardiology, Chang Gung Memorial Hospital, Linkou, Taiwan, ROC

**Keywords:** Pacemaker, Octogenarian, Geriatrics, Survival, Frailty

## Abstract

**Background:**

There is an increased need for permanent pacemaker (PPM) implantation for older patients with multiple comorbidities. The current guidelines recommend that, before implanting PPM, clinicians should discuss life expectancy with patients and their families as part of the decision-making process. However, estimating individual life expectancy is always a challenge.

**Aims:**

We investigated predictors of long-term survival prior to PPM implantation in patients aged 80 or older.

**Methods and results:**

From September 2004 to September 2015, 100 patients aged ≥ 80 years who received PPM implantation were included for retrospective survival analysis. The end point was all-cause mortality. Follow-up duration was 4.0 ± 2.7 years. By the end of the study, 54 patients (54%) had died. Of the 54 who died, 40 patients (74.1%) died of non-cardiac causes. Their survival rates at 1, 2, 3, 5, and 7 years were 90%, 76%, 54%, 32%, and 16%, respectively. Patients with a longer length of hospital stay before PPM implantation (LOS-B) [hazard ratio (HR) 1.03, 95% confidence interval (CI) 1.02–1.05, *p* < 0.001], estimated glomerular filtration rate (eGFR) < 30 ml/min/1.73 m^2^ (HR 4.07, 95% CI 1.95–8.52, *p* < 0.001), body mass index (BMI) < 21 kg/m^2^ (HR 2.50, 95% CI 1.16–5.39, *p* = 0.02), and dyspnea as the major presenting symptom (HR 2.88, 95% CI 1.27–6.55, *p* = 0.01) were associated with lower cumulative survival.

**Conclusions:**

Longer LOS-B, lower eGFR and BMI, and dyspnea as the major presenting symptom are pre-PPM implantation predictors of long-term survival in patients aged 80 or older.

## Introduction

The world population is aging. According to the World Population Prospects reported by the United Nations, the number of people aged 80 or above is projected to triple from 137 million in 2017 to 425 million in 2050 [1]. The aging population results in progressively increasing need for permanent pacemaker (PPM) implantation for older patients with multiple comorbidities [2]. The current guidelines recommend that clinicians discuss the probability of living more than 1 year with patients and their families during decision-making about PPM implantation [3]. However, estimating individual life expectancy before the procedure is always a challenge. Data on pre-PPM implantation predictors of long-term survival in patients aged 80 or older have been limited. Most previous studies on this topic were carried out at least 10 years ago and do not reflect the current situation [4–6]. A landmark study by Udo et al. in 2012 included many important baseline characteristics as possible predictors [7] but lacked clinical data such as symptoms, left-ventricle ejection fraction (LVEF), estimated glomerular filtration rate (eGFR), and length of hospital stay. Recent studies have begun to pay more attention to the relation between survival and these variables in geriatric patients [8–11]. For this study, we hypothesized that these clinical variables prior to PPM implantation predict long-term survival in patients aged 80 or older. A better understanding of their prognosis before PPM implantation is informative for decision-making in the clinical setting.

## Methods

### Patients

This study included patients age ≥ 80 years who met the indications for chronic cardiac pacing [3] and received PPM implantation in Keelung Chang Gung Memorial Hospital from September 2004 to September 2015 for retrospective analysis. The Keelung Chang Gung Memorial Hospital is a 1098-bed, non-profit proprietary, metropolitan hospital. Patients who received cardiac resynchronization therapy, implantable cardioverter defibrillator, or pacemaker generator replacement were excluded. We collected the patients’ clinical characteristics by chart review. These included age, gender, body weight, body height, serum creatinine level, initial major symptoms, indications for PPM implantation, admission to hospital via emergency department (ED) or out-patient department (OPD), length of stay before PPM implantation (LOS-B), and comorbidities, including hypertension, diabetes mellitus, coronary artery disease (CAD), atrial arrhythmia, old cerebral vascular accident, chronic obstructive pulmonary disease, and echocardiography results.

### Follow-up after discharge and major end point

The patients returned to pacemaker OPD 1–2 weeks after discharge. After the first visit, the follow-up intervals were extended from 1–3 months, then to 6 months, depending on the patient’s condition and physician’s decision. On each follow-up visit, physicians assessed wounds, PPM function, the patient’s general health, and adjusted the PPM settings as needed. Survival status and causes of death were obtained from chart review. When patients were lost to follow-up for more than 3 months, we used telephone contact to obtain the reasons for their absence and their vital status. Follow-up was completed on December 15, 2017. The end point was all-cause mortality.

### Definitions

#### CAD

Patients who met any one of the following criteria were defined as having CAD: (1) history of acute myocardial infarction (AMI); prior percutaneous coronary intervention or coronary artery bypass surgery, (2) ≥ 50% coronary artery stenosis documented by invasive coronary angiograms or multislice computed tomography angiography, (3) positive results on noninvasive stress tests, for example, treadmill exercise electrocardiogram or thallium-201 single-photon emission tomography.

#### Atrial arrhythmias

History of atrial fibrillation, atrial flutter, or sustained atrial tachycardia documented on electrocardiography.

#### Echocardiography

All patients received echocardiography examinations during the month preceding PPM implantation. We reviewed the echocardiography results as follows: LVEF was calculated based on Simpson’s method or the M-mode method according to the patient’s clinical condition. Valvular heart disease was defined as a history of valve surgery or at least moderate severity valve dysfunction assessed in accordance with the practice guidelines [12].

#### Estimated glomerular filtration rate (eGFR)

eGFR was calculated using the Modification of Diet in Renal Disease formula: eGFR (ml/min/1.73 m ^2^) = 186.3 × (serum creatinine mg/dl)^−1.154^ × (age) ^−0.203^ × 0.742 (if female). We categorized the eGFR values as > 60, 30–60, and < 30 ml/min/1.73 m^2^, with the upper range based on the definition of chronic kidney disease (CKD) [13]. The lower range was based on the definition of CKD stage IV [13], and the findings of eGFR < 30 were associated with increased risk for all-cause mortality in older patients [14].

#### Body mass index (BMI)

BMI was calculated by dividing body weight in kilograms by the square of body height in meters. BMI values were categorized as > 27, 21–27, and < 21 kg/m^2^. The upper range was based on the Taiwanese definition of obesity [15]. The lower range was based on the population-specific cut-off point for low BMI on the Mini-Nutritional Assessment for older Taiwanese [16].

#### Causes of death

Death due to cardiac causes was defined as death resulting from AMI, congestive heart failure (CHF), ventricular tachycardia, ventricular fibrillation, or death without clear non-cardiac causes. PPM-related death was defined as death related to lead or generator dysfunction, and/or any procedure complications like infection, pneumothorax, or cardiac tamponade.

### Statistical analysis

The data are expressed as the mean ± the standard deviation for normally distributed continuous variables, medians (lower quartile; upper quartile) for skewed variables, and number (percentage) for categorical variables. We used the Cox regression analysis to assess the effects of different variables on survival. The variables with a *p* value of < 0.2 in univariate analysis were included in the multivariate analysis. Hazard ratios (HRs) and 95% confidence intervals (CIs) were also calculated. We assessed the differences in LOS-B between groups using the Mann–Whitney *U* test. The survival curve after PPM implantation was plotted using the Kaplan–Meier method and statistical significance was determined by the log-rank test. A *p* value of < 0.05 was considered statistically significant. Statistical analyses were performed using SPSS Statistics 17.0.

## Results

### Patient characteristics

Of the 230 patients receiving PPM implantation from September 2004 to September 2015, 106 patients (46.1%) were aged 80 and above. Follow-up was completed for 100 (94.3%) of these patients (52 men and 48 women). During follow-up, six patients were lost to contact with vital status unknown and were thus excluded from the following analysis. The age at implantation was 84.5 (81.3; 88.0) years. Most patients were admitted from the ED. The majority of patients presented with dyspnea, followed by dizziness, syncope, or near syncope (Table 1). Low eGFR (< 30 ml/min/1.73 m^2^) and low BMI (< 21 kg/m^2^) were noted in 39 patients (39%) and 17 patients (17%), respectively.

**Table 1:** Baseline characteristics of patients receiving permanent pacemaker implantation aged 80 or over (*n* = 100)

Characteristics	
Follow-up duration (years)	4.0 ± 2.7^a^
Age at implantation (years)	84.5 (81.3; 88.0)^b^
Male gender, *n* (%)	52 (52.0)
Admitted from emergency department, *n* (%)	72 (72.0)
Left-ventricle ejection fractio*n* (%)	68 (62; 73)^b^
Major presenting symptom	
Syncope, near syncope, *n* (%)	24 (24)
Dizziness, *n* (%)	26 (26)
Dyspnea, *n* (%)	50 (50)
Comorbidities	
Hypertension, *n* (%)	88 (88.0)
Diabetes mellitus, *n* (%)	33 (33.0)
Coronary artery disease, *n* (%)	25 (25.0)
Valvular heart disease, *n* (%)	24 (24.0)
Cerebral vascular accident, *n* (%)	23 (23.0)
Chronic obstructive pulmonary disease, *n* (%)	19 (19.0)
Atrial arrhythmia, *n* (%)	39 (39.0)
eGFR (ml/min/1.73 m ^2^)	50.2 (26.2; 66.8)^b^
< 30, *n* (%)	39 (39.0)
30–60, *n* (%)	31 (31.0)
> 60, *n* (%)	30 (30.0)
BMI (kg/m^2^)	24.0 ± 4.1^a^
< 21, *n* (%)	17 (17.0)
21–27, *n* (%)	62 (62.0)
> 27, *n* (%)	21 (21.0)
Indications for implantation	
AVCD, *n* (%)	60 (60.0)
SSS, *n* (%)	30 (30.0)
AFSVR, *n* (%)	10 (10.0)
LOS-B (days)	6 (3; 11)^b^
Dual-chamber pacemaker	80 (80)

eGFR, estimated glomerular filtration rate; BMI, body mass index; AVCD, atrioventricular conduction dysfunction; SSS, sick sinus syndrome; AFSVR, atrial fibrillation with slow ventricular response; LOS-B, length of hospital stay before permanent pacemaker implantation

^a^Mean ± SD

^b^Medians with interquartile range

The patients’ LVEFs were normal. Atrioventricular conduction dysfunction (AVCD) was the most common indication for PPM implantation, followed by sick sinus syndrome (SSS) and atrial fibrillation with slow ventricular response (AFSVR). Atrial arrhythmias were noted in 39 patients (39%). The median of LOS-B was 6 days. Eighty patients (80%) received dual-chamber (DDD or DDDR mode) PPM implantation and the others (20%) received single-chamber (VVI or VVIR mode) PPM implantation. Baseline characteristics are shown in Table 1.

### PPM implantation-related complications and causes of death

PPM implantation-related complications occurred in five patients (5%). No procedure-related mortality occurred. Of the five patients with complications, three developed pocket hematoma, one developed pneumothorax, and one developed a small amount pericardial effusion. Except for the pigtail catheter placement for pneumothorax, all four of the other patients were managed successfully with conservative observation, without the necessity of fluid resuscitation, antibiotic treatment, re-intervention, or any invasive procedure. Despite the complications, all the five patients continued to have stable vital signs. No other significant complication was noted, such as wound or pacing system infection, cardiac tamponade, hemothorax, lead or pulse generator problem, or procedure-related mortality.

The follow-up duration was 4.0 ± 2.7 years, with the longest follow-up of 11.1 years. The survival rates at 1, 2, 3, 5, and 7 years were 90%, 76%, 54%, 32%, and 16%, respectively. By the end of the study, 54 patients (54%) had died. Of the 54 deceased patients, 40 (74.1%) died of non-cardiac causes. The most common non-cardiac cause of death was pneumonia which occurred in 14 patients (35%), followed by 12 with sepsis (30%), 8 with cancer (20%), 5 who suffered cerebral vascular accidents (12.5%), and 1 who experienced trauma (2.5%). Eleven patients (20.4%) died of cardiac causes, and five (45.5%) died of unknown causes despite attempted resuscitation. No deaths were PPM-related. The causes of mortality are shown in Table 2.

**Table 2:** Causes of death in 100 patients aged 80 or older who received permanent pacemaker implantation during follow-up of 4.0 ± 2.7 years

	No.
Non-cardiac causes	40
Pneumonia, *n* (%)	14 (35)
Sepsis, *n* (%)	12 (30)
Cancer, *n* (%)	8 (20)
Cerebral vascular accident, *n* (%)	5 (12.5)
Trauma, *n* (%)	1 (2.5)
Cardiac causes	11
Resuscitation for unknown cause, *n* (%)	5 (45.5)
Acute myocardial infarction, *n* (%)	3 (27.3)
Congestive heart failure, *n* (%)	3 (27.3)
Unknown	3
Permanent pacemaker related	0
Total	54

### Predictors of survival

In Cox univariate analysis, the following variables were significantly associated with lower cumulative survival rate: dyspnea as the major presenting symptom, eGFR < 30 ml/min/1.73 m^2^, BMI < 21 kg/m^2^, AFSVR as the indication for PPM implantation, and longer LOS-B (Table 3). Cox multivariate analysis was performed by including the variables with *p* value < 0.2 from the univariate analysis. Cox multivariate analysis showed that major symptoms, eGFR, BMI, and LOS-B, were independently associated with survival. We found that the following criteria were associated with worse long-term survival: patients with dyspnea as the major presenting symptom (HR 2.88, 95% CI 1.27–6.55, *p* = 0.01), eGFR < 30 ml/min/1.73 m^2^ (HR 4.07, 95% CI 1.95–8.52, *p* < 0.001), BMI < 21 kg/m^2^ (HR 2.50, 95% CI 1.16–5.39, *p* = 0.02) and longer LOS-B (HR 1.03, 95% CI 1.02–1.05, *p* < 0.001). Age was not significantly associated with worse long-term survival (HR 1.06, 95% CI 0.99–1.14, *p* = 0.09) (Table 3).

**Table 3:** Univariate and multivariate analyses for predictors of all-cause mortality in 100 patients aged 80 or over who received permanent pacemaker implantation

Variables	Univariate analysis		Multivariate analysis	
	HR (95% CI)	*p* value	HR (95% CI)	*p* value
Age at implantation^a^	1.05 (0.99–1.11)	0.10	1.06 (0.99–1.14)	0.09
Male gender	0.73 (0.43–1.24)	0.25		
Admitted from ED	1.22 (0.67–2.25)	0.52		
Ejection fraction (%)^b^	0.99 (0.97–1.02)	0.66		
Major presenting symptom		0.09		0.04
Dizziness	Reference		Reference	
Syncope, near syncope	1.59 (0.70–3.64)	0.27	1.90 (0.77–4.71)	0.17
Dyspnea	2.18 (1.07–4.47)	0.03	2.88 (1.27–6.55)	0.01
Hypertension	1.03 (0.50–2.14)	0.93		
Diabetes mellitus	1.37 (0.78–2.42)	0.28		
Coronary artery disease	1.29 (0.69–2.43)	0.43		
Valvular heart disease	1.47 (0.81–2.67)	0.21		
Cerebral vascular accident	1.34 (0.74–2.44)	0.34		
COPD	1.07 (0.52–2.19)	0.87		
Atrial arrhythmia	0.79 (0.45–1.39)	0.42		
eGFR (ml/min/1.73 m ^2^)		0.01		< 0.001
> 60	Reference		Reference	
30–60	1.05 (0.55–1.99)	0.88	1.21 (0.59–2.48)	0.60
< 30	2.56 (1.31–5.00)	0.006	4.07 (1.95–8.52)	< 0.001
BMI (kg/m^2^)		0.04		0.03
21–27	Reference		Reference	
< 21	2.41 (1.19–4.90)	0.01	2.50 (1.16–5.39)	0.02
> 27	1.09 (0.55–2.18)	0.79	0.71 (0.32–1.57)	0.39
Indications for implantation		0.03		0.15
SSS	Reference		Reference	
AVCD	1.89 (0.96–3.74)	0.07	1.11 (0.52–2.39)	0.79
AfSVR	3.42 (1.39–8.40)	0.007	2.31 (0.90–5.90)	0.08
LOS-B (days)^b^	1.03 (1.01–1.04)	< 0.001	1.03 (1.02–1.05)	< 0.001

HR, hazard ratio; CI, confidence interval; ED, emergency department; COPD, chronic obstructive pulmonary disease; eGFR, estimated glomerular filtration rate; BMI, body mass index; SSS, sick sinus syndrome; AVCD, atrioventricular conduction dysfunction; AFSVR, atrial fibrillation with slow ventricular response; LOS-B, length of hospital stay before pacemaker implantation

^a^Per 1 year increase in age

^b^Per unit increase

**Fig. 1:**
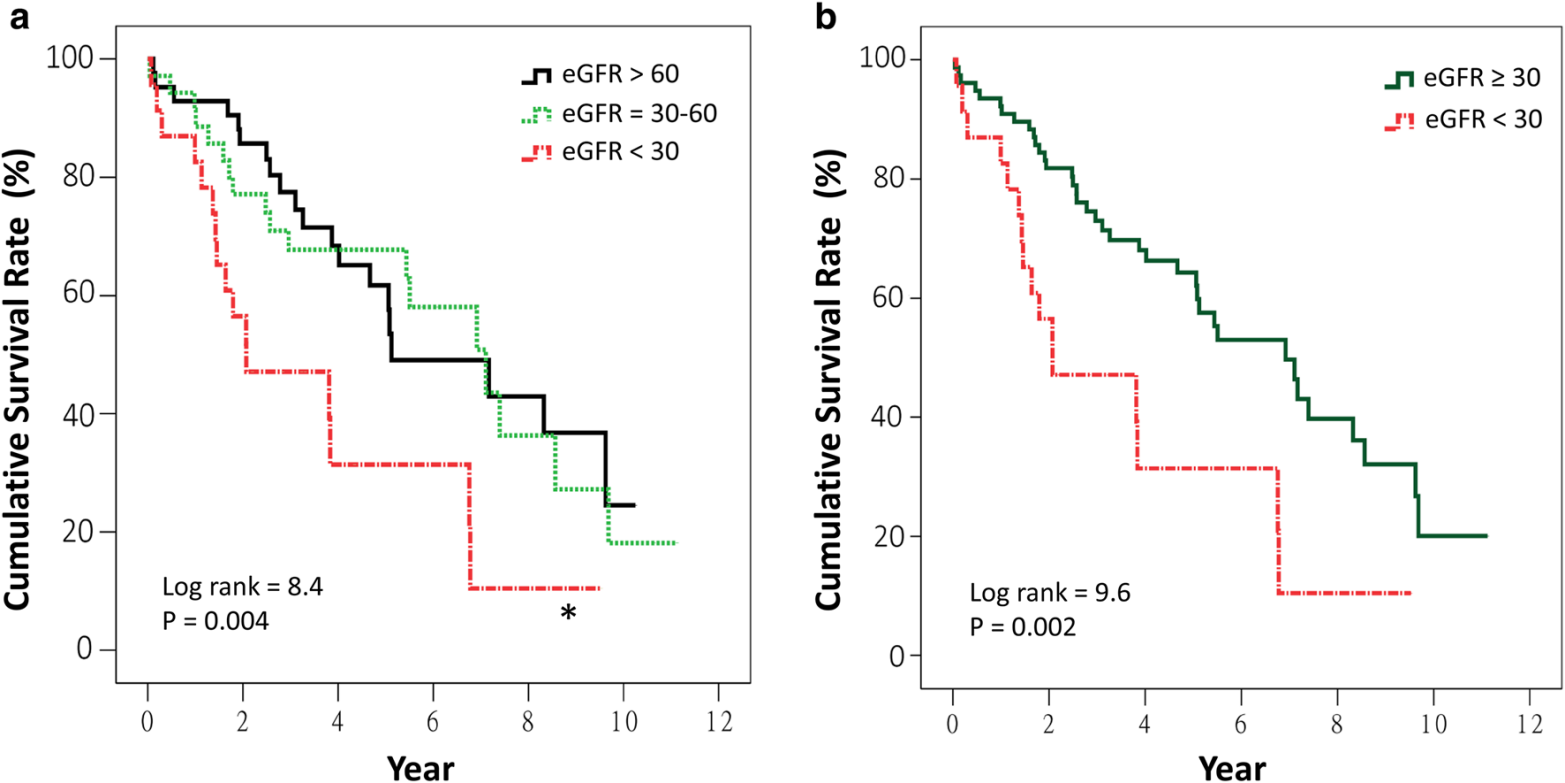
Kaplan–Meier survival curves for all-cause mortality after permanent pacemaker implantation in patients aged 80 or older categorized by the estimated glomerular filtration rates (eGFR) of > 60, 30–60, and < 30 ml/min/1.73 m^2^ (**a**), and categorized by ≥ 30 and < 30 ml/min/1.73 m^2^ (**b**). *p = 0.004, compared to eGFR > 60 ml/min/1.73 m^2^

**Fig. 2:**
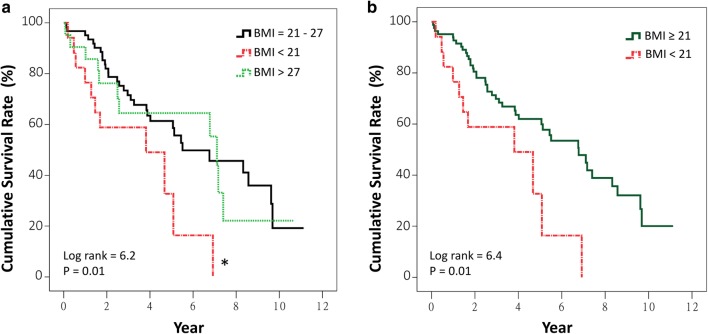
Kaplan–Meier survival curves for all-cause mortality after permanent pacemaker implantation in patients aged 80 or older categorized by body mass index (BMI) < 21, 21–27, and > 27 kg/m^2^ (**a**), and categorized by ≥21 and < 21 kg/m^2^ (**b**). *p = 0.01, compared to BMI = 21–27 kg/m^2^

**Fig. 3:**
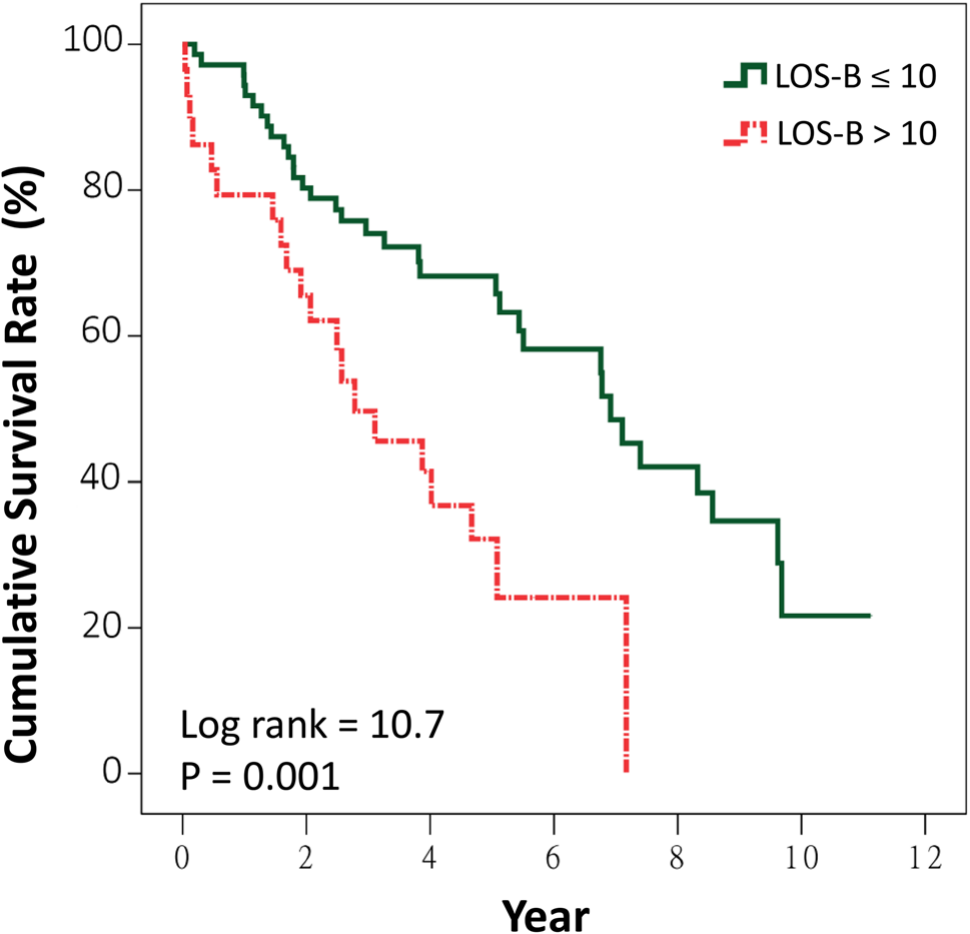
Kaplan–Meier survival curves for all-cause mortality after permanent pacemaker implantation in patients aged 80 or older stratified by the length of hospital stay before permanent pacemaker implantation (LOS-B)

Kaplan–Meier curves reveal that patients with an eGFR < 30 ml/min/1.73 m^2^ had significantly lower cumulative survival compared to those with an eGFR > 60 ml/min/1.73 m^2^ (*p* = 0.004) (Fig. 1a). No significant difference was noted between the curves of eGFR 30–60 and > 60 ml/min/1.73 m^2^. Patients with eGFR < 30 ml/min/1.73 m^2^ had significantly lower cumulative survival compared to others (*p* = 0.002) (Fig. 1b). Patients with BMI of < 21 kg/m^2^ had significantly lower cumulative survival compared to those with BMI of 21–27 kg/m^2^ (*p* = 0.01) (Fig. 2a) and compared to those with BMI ≥ 21 kg/m^2^ (*p* = 0.01) (Fig. 2b). When LOS-B was categorized into ≤ 10 and > 10 days (the third quartile of LOS-B), the median survival duration in patients with an LOS-B of > 10 days was significantly shorter than others (2.8 years, 95% CI 1.2–4.4 years, versus 6.9 years, 95% CI 5.2–8.7 years, *p* = 0.001) (Fig. 3).

### Length of stay before PPM implantation (LOS-B)

Median LOS-B was 6 days. The LOS-B was significantly longer in 35 patients (35%) due to systemic infection with a need for parenteral antibiotic treatment [11 (5; 8) days versus 5 (2; 8) days, *p* < 0.001], in 9 patients (9%) due to respiratory failure with mechanical ventilator support [13 (11; 24) days versus 5 (3; 10) days, *p* < 0.001], in 21 patients (21%) due to acute kidney injury resulting in electrolyte imbalance and fluid overload [13 (9; 20) days versus 5 (3; 9) days, *p* < 0.001], in 12 patients (12%) who needed cardiac catheterization to estimate CAD [10.5 (8; 13.5) days versus 5 (3; 11) days, *p* = 0.006], and in 5 patients (5%) who needed coronary artery intervention for significant CAD [13 (8; 32) days versus 5 (3; 11) days, *p* = 0.02]. The LOS-B was insignificantly longer in seven patients (7%) due to gastrointestinal tract bleeding [8 (3; 10) days versus 6 (3; 11) days, *p* = 0.7].

## Discussion

This study analyzed the pre-PPM implantation predictors of long-term survival in patients aged 80 or older. The main findings of this study are as follows: (1) LOS-B, eGFR, BMI, and dyspnea as the major presenting symptoms were independent predictors of long-term survival. (2) The survival rates were 90%, 76%, 54%, 32%, and 16% at 1, 2, 3, 5, and 7 years, respectively. (3) Most of the patients (74.1%) died of non-cardiac causes.

Previously, Udo et al. performed a study focusing on the long-term outcomes of cardiac pacing in octogenarians and nonagenarians. They found that age at the time of implantation, male gender, congestive heart failure, coronary pathology, and diabetes mellitus were independent predictors of all-cause mortality [7]. However, they did not include informative clinical data such as symptoms, LVEF, eGFR, and the length of hospital stay as potential predictors of long-term survival prior to PPM implantation. We included these potential predictors prior to PPM implantation in our study to further investigate factors predicting long-term survival in patients aged 80 and above.

### Survival after PPM implantation in patients aged 80 or older

Our study revealed that the survival rates after PPM implantation in patients aged 80 or older were 90%, 76%, 54%, 32%, and 16% at 1, 2, 3, 5, and 7 years, respectively. Most of the patients (74.1%) died of non-cardiac causes. These findings are similar to the results obtained by Udo et al., which showed 86%, 75%, and 49% at 1, 2, and 5 years, respectively; 69.8% of those patients died of non-cardiac causes [7].

The duration of time from the initial implantation to replacing a PPM generator varied between 6 and 7 years in different studies [17, 18]. In our study, survival duration was from less than 1 year to longer than 10 years. Actually, 16% of patients remained alive 7 years after PPM implantation. This implies that, with proper care, some of very old patients can still survive for a significantly long period of years. Therefore, correctly identifying them before PPM implantation and optimizing the PPM program settings during follow-up were essential to enhance PPM longevity and avoid repeated generator replacement. On the other hand, there were 10% of patients died within 1 year after PPM implantation. Whether or not to proceed with PPM implantation in these patients is not only a medical or economic issue but also an ethical one. Our findings suggested that, for patients aged 80 and above with the following clinical characteristics: longer LOS-B, eGFR < 30 ml/min/1.73 m^2^, BMI < 21 kg/m^2^, or dyspnea as the major presenting symptom, comprehensive geriatric assessment should be considered to perform before PPM implantation with the purpose of planning and/or delivering medical, psychosocial, and rehabilitative care [19]. In addition to identifying these patients early, decision-making should be shared with patients and their families. Interventions like participating in a rehabilitation program and a better nutritional support may also be helpful for this population at risk.

### LOS-B as a predictor of long-term survival

Our study suggested that longer LOS-B was associated with shorter long-term survival. Although the mechanisms are not entirely clear, longer LOS-B may be one indicator of frail status, which predicts poor long-term survival. Furthermore, we found that causes of prolonged LOS-B were substantially associated with a variety of coexisting multiple systemic diseases. It is not uncommon to encounter such complex conditions in clinical practice. When hospitalized, geriatric patients often present with active problems in multiple organs due to compromised homeostatic mechanisms as a result of aging-associated decline in functional reserve across multiple physiological systems [20]. Multiple comorbidities resulted in prolonged length of hospital stay. The previous studies have already reported that frailty and prolonged length of hospital stay in frail patients [11, 21, 22] are associated with poor long-term survival [23, 24].

To the best of our knowledge, no previous study has addressed the relation between length of stay before pacemaker implantation (LOS-B) and long-term survival. Most prior studies of geriatric patients receiving PPM implants did not include data on LOS-B [4–7, 25, 26], or only noted the total length of hospital stay [27] or length of stay after the procedure [28]. Since multiple organ problems often delay PPM implantation, LOS-B could be a practical indicator for predicting long-term survival in geriatric patients before the procedure.

### The eGFR as a predictor of long-term survival

Our study results suggested that eGFR < 30 ml/min/1.73 m^2^ before PPM implantation is a predictor of worse outcomes among patients aged 80 and above. CKD was associated with multiple comorbidities [29]. In addition, CKD not only correlated with functional limitations [30], but also with greater likelihood of frailty, which itself predicts poor survival [14, 31]. There is increasing recognition that CKD is associated with increased all-cause mortality in specific populations [14, 32–34]. To the best of our knowledge, no previous study has focused on the association between eGFR and long-term survival in patients receiving PPM implantation. A better understanding of patients’ eGFR before PPM implantation can be helpful for predicting prognosis.

### BMI as a predictor of long-term survival

In line with the previous studies in general or older populations, we found that being underweight is associated with higher mortality [35–38]. For the aged, being underweight might signal loss of appetite, poor nutrition, and/or poor emotional well-being. These could lead to limited daily activity, sarcopenia, frailty, and increased risk of mortality [39, 40].

Another finding of the study is that BMI > 27 kg/m^2^ was not associated with worse long-term survival. The relation between overweight-obesity and mortality in older people remains controversial and has been described with either U- or L-shaped curves. The U-shaped curves represent significantly increased mortality observed both in underweight and overweight-obese people [35]. The L-shaped curves represent significantly increased mortality only among the underweight, but not among the overweight-obese people [36–38, 41]. This is referred to as the obesity paradox [42]. Theoretically, the L-shaped curves are expected to occur more frequently in older study populations in whom obesity-related diseases like cardiovascular or cerebrovascular diseases are not the leading cause of mortality [37]. Our study focused on very old patients whose leading causes of mortality that we found to be non-cardiovascular disease. The obesity paradox does appear in our study cohort.

Only one previous study has addressed BMI and long-term survival in older patients receiving PPM implantation. In contrast to our results, the study by Udo et al. showed no association between BMI and long-term survival [7]. Since the previous studies on non-PPM recipients suggested a non-linear relation between BMI and survival [35–38, 41], the negative findings by Udo et al. might be due to different ethnicities of the patients or to their study design, which did not divide BMI into different categories.

### Dyspnea as a predictor of long-term survival

Our study found that patients with dyspnea as the major initial symptom were associated with worse long-term survival compared with those who presented with symptoms of dizziness, pre-syncope, or syncope. Recent studies have suggested that dyspnea is correlated with cardiopulmonary and physical performance impairments, and could be a potential marker of frailty in adults. It is not only a symptom but also associated with increased mortality from all causes [8, 43]. Although bradycardia may cause a variety of signs and symptoms, exercise intolerance may indicate advanced dysregulation in response to bradycardia. Very few previous studies have included the symptom of dyspnea as a potential predictor for long-term survival in aged patients receiving PPM implantation [6]. Our study suggested that dyspnea is not only a symptom of bradycardia, but also it might have important prognostic implications for long-term survival in older patients receiving PPM implantation.

### Study limitations

This is a single hospital retrospective study and we could not exclude selection-bias. The relatively small sample size also reduces the power of the study. Moreover, due to limited data available through chart review, we did not include psychosocial and functional assessments, which are important components of comprehensive geriatric assessment. Furthermore, our study focused on survival duration but did not mention quality of life, which is also an important issue. The results presented here should be confirmed by prospective, large-scale randomized-controlled trials with long-term follow-up.

## Conclusions

Our study shows the cumulative 3-year survival rate after PPM implantation in patients aged 80 or older was 54%. Survival duration varied from less than 1 year to longer than 10 years. Three-fourths of the patients died of non-cardiac causes. Longer LOS-B, eGFR < 30 ml/min/1.73 m^2^, BMI < 21 kg/m^2^, and dyspnea as the major presenting symptom predicted worse long-term survival. Assessment of these risk factors before PPM implantation is informative for clinical practice and decision-making.
